# Dr. Dewees's Examination of Dr. Osborn's Opinion of the Physical Necessity of Pain and Difficulty in Human Parturition

**Published:** 1807-12

**Authors:** 


### Dr. DEWEES's *Examination of* Dr. OSBORN's *Opinion of the Physical Necessity of Pain and Difficulty in Human Parturition.*

[ Continued from our last Number, pp. 450—456, ]

IF it should be asked why pain occurs, for the most part, in labours that are so rapid as to employ but a few minutes? I would answer,

That the uterus possesses two distinct kinds of actions; the one regular and constant, and always tending to diminish its capacity, when its sides are distracted, or when the distracting force is withdrawn; it is capable of occasional and powerful augmentation; and, in a natural and unperverted state, is sufficient to effect the delivery of the child. Of this kind, is that action which reduces the uterus to its original bulk after delivery; of this kind, is that action which effects delivery among females in a savage state, and among those of Calabria, &c.; of this kind, is that action of the uteri of brutes, which relieves them of their burthen; of this kind, is that effort which expels the child after visible life has ceased in the mother*: this kind of action, or contraction of the uterus, is not attended with pain. This is called the tonic contraction of the uterus. The other action of the uterus is a spasmodic one, and attended with pain. This is a distinct action from the other; and in this instance dependent on it.

This last kind, or spasmodic contraction of the uterus, I am disposed to consider, for the most part, if not altogether, artificial or accidental to women; my reasons for thinking so are,

---

* Harvey, Baudelocque, &c.

First.

First. No physical or absolute necessity for pain ever has been, or ever can be, demonstrated.

Secondly. Women in a state of nature are, for the most part, exempt from it.

Thirdly. If analogy will be allowed to be called in, I can urge, the exemption of brute animals from it, though possessing very similar conformation of pelvis, &c. to the human.

Fourthly. Many women, among those who, for the most part, have pain in their labours, are sometimes free from it.

Fifthly. If not being essential to delivery, as children have been born after the death of their mothers, by the tonic contraction of the uterus alone; and many women have pains in various parts of their bodies independent of the uterus; as in the jaws, head, knees, &c.

Pain is in very various proportions among women who are equally well formed; we generally find the women of the country more obnoxious to it, than those of cities; and the hard-working or laborious part of those in cities, more afflicted than those who live more luxuriously and indolently. Various reasons might be assigned for this difference; I shall however only observe, that, wherever we find that state of fibre which is termed rigid, we shall there find also, cæteris paribus, more pain during labour; with this state of fibre there appears to be connected, (or it may exist in this very state) a greater disposition in the system in general, or in the uterus in particular, to take on what is termed, inflammatory action;—and hence the utility of blood-letting, and that sometimes to a great extent, in those labours that are attended with rigid os uteri, or unyielding external parts. I have frequently seen this remedy act like a charm; it not only hastens the labour, by diminishing the resistance of the soft parts, but also, by the same means, abates pain, as there is now a lesser obstacle to overcome.

From what has been said, it would appear that the general effects of society and refinement, have produced certain changes on the human female constitution; and that these changes have produced their consequences; which consequences have given rise and continuance to pain and difficulty in human parturition.

I will now attend to Dr. O. when he considers "the peculiarities of the quadruped, and their operation in labour."

"By the horizontal position of the quadruped, the parietes rietes of the abdomen support the gravid uterus during gestation, in whatever situation the animal may be; the parts concerned in labour cannot, therefore, at any time, be exposed to the general influence of gravity; on which account, nature was not required to observe such strict laws, or be attentive to such minute deviations, respecting either the position, or capacity of the pelvis, the volume, or form of the head of the fœtus, the situation or structure of the soft parts. Therefore the same, or very nearly the same axis is given the trunk, the pelvis, the vagina, and the os externum; nature has likewise made the head proportionably small, compared with the capacity of the pelvis; so that it may readily pass through in any direction; and the soft parts, having nothing to support, are of a loose texture, easily yielding to the pressure of the membranes, or fœtus, and of course affording little resistance, and no impediment to delivery."

From the horizontal position of the quadruped pelvis, nearly the same consequences result, as in the human female; for the fœtus in them neither will, nor can be made, to engage in the pelvis, until forced by the contractions of the uterus; this is precisely the case in women: and however widely the axis of the uterus, and that of the superior strait may differ before labour, we find a perfect correspondence immediately after, and this is all that is required.

Dr. O. supposes a great degree of rigidity necessary in the uterus, in order that it may support the fœtus; and that this is one of the causes of difficulty in human parturition; but it can be readily demonstrated it is not necessary, even by his own words.

The head, he says, cannot engage by its gravity, since the axis of the uterus and the superior strait are not the same. If this be true, how can the head be exactly over the opening of the pelvis; and if it be not exactly over the opening of the pelvis, it must impinge on some portion of its margin; and if it does impinge on a portion of the margin of the pelvis, what great weight can the uterus have to support? Thus then we see, rigidity is not absolutely necessary; consequently, must not be considered as a cause, naturally productive of pain and difficulty.

Dr. O. admits, however, that the axes of the uterus, pelvis, and vagina, are not exactly the same in the quadruped: to what is the power given to make them correspond? It can only be to the uterus: and has not the human man female the same agent? and does it not perform its duties equally well?

"The head of the brute fœtus," he says, "is proportionably small, compared with the capacity of the pelvis, so that it may readily pass through in any direction;"—but upon a strict examination, it will be found to bear the same relation to the different diameters of the pelvis, as the head of the human fœtus does to its pelvis; and in the latter, it is a well-known fact to accoucheurs, that, it might in general be larger without producing an increase of difficulty; but the Doctor certainly labours under an error, when he says, that the head of the quadruped fœtus may easily pass in any direction; since it is neither consonant with the structure of the parts, nor the mechanism of labour, as daily experience proves.

The soft parts in the brute, he says, "are of a loose texture, easily yielding to the first pressure of the membranes, or fœtus, and of course affording little resistance and no impediment to delivery." But the Doctor admits they dilate; so they do in the human subject, and in a state of nature with as much facility; and this is all that is necessary.

Dr. O. asserts, that it is from this peculiar structure of the soft parts of brute animals, that a laceration of the perinæum never happens; and also, that it is owing to the rigid structure of the human subject, that it frequently happens with them.

These assertions of Dr. O. like some others already noticed, are rather unqualified, since it has happened with the cow and the mare, as I myself have witnessed, either from the extraordinary size of the fœtus, or its bad situation. That it is not an unfrequent accident in human parturition, is admitted; but it is the effect, in general, of ignorace or inattention, agreeably to the Doctor's own confession. But if it be admitted that this takes place in human parturition, even under the most cautious management, (for Dr. Denman has acknowledged it having happened under his care) what does it prove? most assuredly not what Dr. O. wishes; on the contrary, it is another support to the position, that the changes produced on the female constitution by civilization and refinement, are the causes of pain and difficulty in human parturition; and that this artificial state of the perinæum, whereby it is endangered from the passage of the child, is a corroboration; for who is there to guard the perinæum of the American rican squaw, or the wandering Arab? yet we hear not of this accident among them.

After following Dr. O. through his principal arguments, it will be proper to advert, for a moment, to his conclusion.

"By this sketch of human and comparative parturition, it is evident, why this operation, under the most favourable circumstances, or natural labour, must in women be attended with much more pain, difficulty, and delay, than in any other creature."

Here we find the Doctor again asserting more than he has proved; for if his words, "under the most favourable circumstances," mean any thing, they must imply—that human parturition must, under all or any other circumstances, be attended with pain, difficulty, and delay; this, I trust, has already been shewn, is not the case.

The Doctor then adds, "it remains now to be explained, why laborious parturition never did, or can occur, in the quadruped."

"It is well known that the great and genuine cause of difficult and laborious births, is the deformity of the pelvis, or the disproportion of its cavity to the volume of the child's head; and that this deformity is caused by a disease peculiar to the human subject, called in infancy rachitis, and in the adult state mollities ossium." Dr. O. might have gone farther and have said, that this disease is not only peculiar to the human subject, but to them only under particular circumstances; he has mistaken a consequence for a cause, and by doing this, he has deserted his original positions; namely, that the erect form of the human female, and her peculiar form of pelvis, (independent of disease, and the inflicted curse) were the physical causes of pain and difficulty in her labours. In attributing pain and difficulty to rickets, he appears to forget there are other causes for them; and from its being a disease peculiar to man, draws a most unwarrantable conclusion, "that laborious parturition never did, nor can occur, in the quadruped."

Dr. O's argument then stands fairly thus: the cause of pain and difficulty in human parturition, is rickets; rickets is peculiar to the human subject, therefore, no other animal can have a painful or laborious labour:—or thus, no animal that is not subject to rickets, can have a laborious or painful labour; no quadruped is subject to the rickets, therefore no quadruped can have a painful or laborious labour.

The absurdity of these premises and conclusions are too glaring to need further refutation. I grant, and I believe it universally obtains, that the quadruped is not subject to the disease just spoken of, but I will by no means agree that this exempts them from painful and laborious births; they are by it only freed from this cause of them, for I have seen, the cow particularly, in extreme agony many hours, from a bad position of the head, notwithstanding Dr. C's bold assertion, "that it is proportionably small, and may pass in any direction."

From what has been said, I trust, it has been made appear, that pain and difficulty are not physically necessary.

